# Ups and downs of drug rehab among women: a qualitative study

**DOI:** 10.1186/s12905-020-00946-2

**Published:** 2020-04-22

**Authors:** Zahra Boroumandfar, Masoud Kianpour, Maryam Afshari

**Affiliations:** 1 Midwifery & reproductive health department, Nursing and Midwifery Care Research Center, Isfahan University of Medical Sciences, Isfahan, Iran; 2 Department of Social Sciences, University of Isfahan, Isfahan, Iran; 3 University of Isfahan, Isfahan, Iran

**Keywords:** Drug use, Lived experience, Substance-related disorders, Women’s health, Qualitative study

## Abstract

**Background:**

According to recent studies, the number of women drug users is dramatically increasing. However, the information on the issue of drug rehab in women is not sufficient, and there are numerous traditional, organizational, political and cultural barriers to the provision of relevant information in this regard in Iran. This study, thus, aimed to explain the factors influencing the decision of these women to stop drug use.

**Methods:**

This qualitative study was conducted in two rehab camps of Isfahan (in Iran) on July to October 2017. Thirty participants (women drug users) were selected through purposive and theoretical sampling until data saturation was reached. Data collection was conducted through semi-structured interviews. The transcribed interviews were analyzed using conventional content analysis.

**Results:**

Based on the analysis of the obtained results, the women’s experience of the ups and downs of stopping drug use yielded two themes and nine sub-themes. The themes were “the need for emancipation (the deviated path, being abused, compulsive drug use, acquaintance with God, a supportive family)” and “Sinking factors (non-assisting mates, pro-addictive family, unawareness of assisting official organization and non-government organization, woman’s lack of authority, ineffective opportunities)”.

**Conclusions:**

It was concluded that addiction rehab strategies can lead to a brighter life for women drug users only when they are coupled with open-hearted assistance of the families and women specific rehab centers are established to help them meet their specific needs.

## Introduction

Substance-related disorders are among the disorders which have much personal, familial and social harm associated with increased tension in the family and abusive behaviors [1]. There are 230 million drug users all around the world, accounting for about 5% of the world’s population [2]. There is no clear report about the precise number of drug users in Iran. Reviewing various domestic sources, Sarrami (2013) reported officially that there are about 1,200,000, drug users in Iran; however, according to the report of unofficial sources by various experts and officials, this number is up to 5 million. The significant point in this regard is that the current situation and the process of drug use in Iran are alarming and deteriorating [3]. As the classic form of drug use consists of a male pattern, drug use is more a masculine than a feminine problem [4]. However, recent studies indicate that the number of women drug users is growing. The proportion of women drug users is estimated to range from 10% in Asian countries to 40% in European countries [5].

The results of a survey in Shiraz, in the southern part of Iran, showed that about 3% of the subjects had used heroin at least once in their lifetime [6]. In the 1970s, 4.5% of the drug users were estimated to be women; however, this ratio has increased to 10% according to the official statistics in Iran [7]. Because of the factors such as stress, negative mood in relationships, turbulent and violent family environment, addicted spouses, psychiatric illnesses and sexual violence in women, they are more likely than men to be involved with drug use [8]. Khademian (2008) stated in a study that disruption in the conjugal life of husband and wife, disintegration of the parental life, problems in the conjugal life of the couples, socioeconomic problems, drug availability, and family members’ drug use are among the factors affecting the use drug in Iranian women [9]. Although drug use is less common in women than in men, it has higher medical, social and economic consequences in women. These consequences include unwanted pregnancy, trauma, violence and transmission of infectious diseases such as HIV (human immunodeficiency viruses) and hepatitis B and C [10]. Additionally, social harms such as runaway, prostitution, and high-risk behaviors are among the harms associated with drug use in women [7] which endanger their health and challenge their rehabilitation process [11]. Various studies have identified factors effective in the decision of the drug users to stop drug use. Negative attitudes toward women drug users, lack of family and friends support (compared to the male ones), economic poverty, redundant bureaucracies, the focus of rehab programs on men, and the lack of specialist rehab centers for these women are among the barriers to the rehabilitation of them [11]. McDonnell (2010) showed in a study that the most important factors in finding a way for drug detoxification include the drug users’ understanding of the negative effects of drug use on themselves and others, social deprivations, drug-related stigmatization, imposed heavy costs, negative impact of drug use on the communication with others and family members, inability in doing their job and duties, lack of control over life, and the incidence of physical, mental and social illnesses which make drug users think about the problem and try to stop drug use [12].

The results of the study by Herd (2009) showed that being aroused and tempted to use drug and the motivation and hope to stop drug after using it, together with the lack of self-efficacy and self-esteem are among the important factors of relapsing into drug use [13]. Moreover, labeling is one of the factors contributing to the relapse of drug use. In addition, factors such as addicted friends, psychological stress, referring to the former locations or hangouts, unpleasant situations, being rejected by the family and society, and seeing the tools and objects of drug use are other factors in this regard [14]. Studies have shown that religion and its related variables decrease suicidal behaviors [15] and drug use [16]. Unfortunately, numerous studies have shown that there is a high likelihood of relapse into substance use after stopping it. Some studies show that the relapse of drug use has occurred within the first 6 months in 80% of those who have referred to rehabilitation centers to stop drug use [17]. According to the available statistics, drug users refer to rehabilitation centers more than 2 to 3 times, suggesting that the rate of relapse is dramatically high [18]. Accordingly, it seems that more accurate, broader and deeper preventive and therapeutic interventions should be considered for these people in the society. This will not happen unless we identify the factors associated with the treatment of drug users as well as all of the factors effective in this regard and present them in an accessible format. But the society does not have the understanding and capacity to provide services for women drug users, and these women are seen as abusive individuals [19]. Although numerous studies have been conducted on drug-dependent subjects and drug-dependence as a whole, most of them have not considered women as their subject [20]. The growing number of drug-dependent women is one of the important social issues in Iran. Drug rehabilitation has been an issue of concern for authorities, drug users themselves and their families as well. This issue is more important especially in developing and young countries, including Iran, which need more efficient and effective human resources [21]. Although many studies have been conducted on drug use and drug rehabilitation all around the world and in Iran as well [22, 11], less attention has been paid to the decision-making process in this regard. An individual’s attitudes, behaviors, and values construct an important part of the change process [14]. Therefore, the present study was conducted with a qualitative approach in order to develop a deep understanding of the challenges and opportunities of women drug users in their decision to stop drug use through using their experiences. This study thus aimed to explain the factors influencing the decision of these women to stop drug use.

## Methods

### Setting

This qualitative study was conducted in two rehab camps of Isfahan in July to October 2017. Isfahan is located almost in the center of Iran. Isfahan is the third most populous city after Tehran and Mashhad, with an estimated urban population of 2,243,249, 49% of which are women. The people of Isfahan also have a traditional religious culture. Isfahan has two drug rehabilitation camps specifically for women drug users, which are located in the outskirts of the town. Women, who come to these centers for drug rehabilitation, can stay there for 21 days and have to pay for it. No medication is allowed during these 21 days and they can meet only a counselor or other drug users. These women are often self-proclaimed and coming from their families, law enforcement or friends. After leaving the camp, there are no coherent and appropriate managed services to help the sustainability of drug rehabilitation. These two camps are for women drug users, covered and supervised by the Welfare Organization. The researcher has been able to conduct interviews at these two sites by introducing herself through a written letter and following difficult administrative procedures.

### Sampling

The participants were selected by using purposive sampling method from 30 women who had drug use related experiences. Inclusion criteria were having experience of being in a drug rehabilitation process, being volunteer to participate in the study, aged 18 to 50, and having the ability to express their experiences. Exclusion criteria included having any known mental illness (Based on the counselor’s view in rehab camps), the inability of the participant to take part in the interview and answer the researcher’s questions, and having sensory, hearing and speech impairments that might affect the process of the interview and data collection. All 30 participants were interviewed by the first and third authors through a semi-structured interview in the Camp Consultant Office in a quiet and private environment. Besides the selection criteria, we aimed to obtain a diverse range of participants in terms of age, marital status, educational level, and socioeconomic condition.

### Data collection

After selecting the participants according to the study’s inclusion criteria and obtaining the consent of the interviewees, the time and place of the interview was determined. Before the interview, the participants’ permission and consent was obtained for recording the interview. The interviews were recorded by a voice recorder. The voice recorder was placed in front of the participants and, before recording their voice, their permission was obtained. Then, they were asked to identify themselves with a pseudonym. The researcher conducted deep face-to-face and semi-structured interviews with the participants and each interview began with a guiding question. At the beginning of the interview, certain questions were asked so that the researcher could become familiar with the interviewees, gain their confidence, create a safe and relaxed atmosphere, and gain as much information as possible about their personality. Then, open-ended questions like “under what conditions did you start substance use?” “Why did you stop using drugs?” “What has been your experience with drug rehabilitation?” were asked. The next follow-up questions were made based on the participants’ explained experiences. Questions such as “what do you mean?” “Please explain more.” “ Did I get what you mean correctly?” were used to deepen the interviews. All of the questions were designed to deepen the level of understanding. The duration of the interviews ranged between 45 to 60 min based on the physical and psychological conditions of each subject and continued until data saturation was reached. In order for more intimacy to be brought about, the first and third authors spent time in the camps with the subjects before and after each interview. They ate with the participants for a few days and talked about a common topic other than drug use.

### Data analysis

Conventional content analysis [23] of the transcribed interviews and field notes was performed to identify key themes, first by each of us separately and then in joint discussions. During analysis, we avoided preconceived categories, instead allowing the categories to flow inductively from the data. After conducting the interviews, all of the audio files were transcribed and typed verbatim from an audio digital recorder. Next, the transcripts were classified into categories of sentences or paragraphs that were, then, changed to meaning units. We took notes listing our first impressions and thoughts and, then, conceive labels for codes used as an initial coding scheme. Then, the meaning units were collected, summarized, and coded. We then sorted the codes into categories based on relations and links among them. This step was repeated several times until the semantic units extracted from the texts can cover all experiences of the participants with regard to the rehabilitation process and its challenges. Finally, the compilation of the themes was performed to draw the hidden content of the text. We believe that although the described sample was not large, it was unique and might be considered meaningful. When data saturation was reached, sampling was discontinued. In fact, according to the conventional analysis of the qualitative data, when the data in open coding are similar and no new data is found, the interview is terminated and data saturation is reached. The process of data analysis was repeated after each interview, and codes and categories were modified if necessary. The credibility of the data was established by the researchers as a peer check. The data were coded and categorized independently by the first and second authors and, then, the emerging themes were compared. When the authors disagreed, clarifications and discussions continued until a consensus was achieved. We emphasized 80% agreement on the codes. Common eligibility criteria of qualitative research such as verification, reliability and transferability were taken into account in the study through techniques. Therefore, a summary of the interviews was returned to the participants as a member check to confirm that the researcher represented their ideas. Moreover, trustworthiness of the research was established through prolonged engagement with data, constant comparison analysis, and maximum variation of sampling, systematic data collection, quick prescription, using the participants’ views, and reviewing all of the data. Transferability of the research was also confirmed through interviewing various participants.

## Result

Thirty participants attended the study. Their mean age was 30 ± 5 years. Six participants were illiterate (20%), 15 (50%) primary school, and 5 (16.6%) diploma certificate. 15 (50%) were married, 10 (30%) were divorced and 5 (16.6%) were single. Two obtained categories were “the need for emancipation” and “sinking factors”. The categories led to the development of the main theme of "Ups and downs of drug rehab, experienced by the women drug users. Table 1 shows the inductive process of reaching the categories of this study.

**Table 1:** The formation of subcategories, categories and main theme

Category	Subcategories	coded	Meaning units
**The need for emancipation**	The deviated path:	Feel the damage to myself and those around me	Drug made me homeless, that is, I could no longer live, die or even make up my mind. I committed suicide four times. I hated myself; do you know what I’m saying? I had reached a dead end
	Being abused	Physical, psychological and sexual abuse reasons for abandoning substance use	After some days of homelessness, a drug dealer trapped me and I lived in a Sexual hangout for 6 months, and then, I got hepatitis B and genital herpes. I was homelessness and Helpless. I was beaten and raped in the hangout. Everybody abused me. I saw no one was thinking of me and I was going to save myself
	Compulsion to drug abuse	Constant intellectual involvement in the preparation and consumption of drug	There was a state of coercion inside of me that made me use drug, I mean, when we got up, in the mornings I couldn’t accept those days as a human being
	Acquaintance with God:	Thank God it is a practice to return to life	.... I wish that after 10 times in the rehab camps, I wasn’t tempted again. These wishes have been memories for years; and with those same wishes, I will go again to stop drug. God’s remorse is my hope for perseverance
	A supportive family:	Feeling valued through family support for quitting drugs	At my first course (in the rehab camp) my dad and family came to meet me. I kissed my parent’s hands for the first time. They told me we want to keep you here for two courses because we love you. Oh, they told me they loved me..
**Sinking factors**	Non-assisting mates	lacked family support,	“My family did not trust me anymore. I used drugs, I harmed everyone, I destroyed everything; but they still loved me. They did not help me to quit because they could force me. They took me wherever they wanted. They did everything to me: physical abuse, sexual abuse, rational and mental abuse.”
	Pro-addictive family	The availability and the use of drugs by all family members at home	“I didn’t know what to do. Drugs were found at my mom’s home, and also at my dad’s home, or my sister’s. When I went to my home, my husband used drugs in front of me. I had to use, too. I could not refrain, I had a big temptation. I did not want to use it, but I used while crying.”
	unawareness of assisting official organization and non-government organization	Lack of awareness of existing society support services or lack of special rehab centers for women	“I was exhausted. Until 1 day I saw a program about rehab centers of the TV. I thought that these centers were for men only. It was very late for me. Now that I think, I see that if I went to the rehab camp only on opium, the desired result would have come very sooner than when I used several drugs.”
	Woman’s lack of authority	living as a woman under the supervision of a father, husband, brother or partner	“After 20 times that my husband was taken to the rehab camp, my dad once brought me there to quit. However, my husband gave him 400.000 Tomans to bring me back. My husband wanted me to be an addict, so that I may not think of divorce or making him quit drugs. He gave me subsistence and I had no options for quitting. I was a woman and needed food and a shelter.... ”
	Ineffective opportunities	high costs, lack of permission to leave or reenter the camp, not meeting their children, the absence of a doctor or counselor, lack of physical space and adequate food, and sometimes violent	“I’m already a postmenopausal woman. I have hypertension and diabetes. I have to take my medicines. But here they did not allow me to my take them. One of the women had a bad cold and they did not allow her to take an acetaminophen. They take you out only if you die. It is my last time here.”

### A. The need for emancipation

Research has shown that one of the main categories is the “need for emancipation,” which consists of five sub-categories: “the deviated path, being abused, compulsive drug use, acquaintance with God, a supportive family”. Fear of major changes in lifestyle, fear of complications of physical deprivation, fear of criticism and rejection because of drug use, fear of losing the current status, fear of failure in rehab, embarrassment, destruction of the inner religious beliefs, and fear of family pressure to continue the emancipation path were among the threatening factors in the participants for making the decision to rehabilitate. Participant No. 24 stated, for example:

“I decided to put it (drug use) away. Admittedly, I was accustomed to this kind of life, and when I decided to change it, I was really alone. My husband used drug too. He didn’t pay any attention to me. So, I had to solve it myself and decided to come to the rehab camp.”

The deviated path: the conditions that led to being fed up of the status quo, and reaching a dead end were noticeable with subcategories such as feeling worthless, not realizing the realities of life, the lack of attraction to life and dissatisfaction with it, the worthlessness of the drugs, the feeling of being harmful for others (the family), the numerous rehabs and its consequences, physical complications of the drugs and the risk of being rejected by the family. In this regard, participant no. 8 stated:

“… When I look back I see that I sacrificed two lives for drugs. I did not see my children and my husband at all. I was a black stain...When I refrained, I had a runny nose; I had pain in my body and hands. I could not move at all, I was very nervous and aggressive. I was uncontrollable; I was at the end of my rope. I felt the real danger that I may be rejected by my parents. I felt that it was the time. I really had to stop drug. I had to discontinue drug use.”

Abnormal behaviors for acquiring drugs, like prostitution, theft, lying and the resulting psychological and social consequences such as pregnancy, runaway, self-harm and suicide were among the factors influencing the sense of dissatisfaction from the current situation and the decision to rehabilitate. Participant no. 18 said:

“I robbed and was involved in prostitution to gain money for the drugs. I was a rough sleeper. I said to myself: I cannot live like this. I have to die or find a way out. I harmed myself. I have committed suicide four times, I hated myself. You know what I’m saying? I’ve been at the end. It was so late for me that I never even got to the middle of it.”

Being abused: Most of the participants pointed to their physical and emotional condition and being sexually abused. In this path of injustice, they decided to get rid of drug abuse. And save themselves. In this regard, participant no. 3 maintained:

“My husband was sentenced to prison for drug dealing and Welfare Organization took away my children when I was down. Finally, my mother-in-law kicked me out of home. After some days of homelessness, a drug dealer trapped me and I lived in a sexual and drug use hangout for six months, I was beaten several times and raped in the hangout and, then, I got hepatitis B and genital herpes. I was homeless and helpless. But everybody abused me. I felt no one cared about me and I was going to save myself. And I came to the camp to get rid of methamphetamine use.”

Compulsive drug use: For the participants of this study, factors such as compulsion, persistent obsession with the preparation, storage and usage of drugs, and attempt to procure drugs by violating ethical and legal norms provided the conditions for their exhaustion. Participant no. 4, who had a history of drug injection, said:

“Opium was no longer effective. I got into cracks, heroin and crystal (methamphetamine). Eventually, these didn’t work either, and I got into injection. My body did not respond anymore. I was permanently drinking, smoking or injecting. That made me think that it’s the time to stop drug use. It was enough. I was tired of it.”

Acquaintance with God: At the pinnacle of helplessness, “God’s grace” was a resort for returning to life, as participant no. 21 put it:

“I liked to get rid of the fear of rape by men who came to my home to give me drugs. I wish my mother did not leave me alone that day in the garden to get drugs. I wish that after 10 times in the rehab camps, I wasn’t tempted again. These wishes have been memories for years; and with those same wishes, I will go again to drug rehabilitation. God’s grace is my hope for perseverance.”

A supportive family: Some of the participants feel good about having family support and encouragement. It is impossible to deal with problems in the physical, mental, therapeutic, and financial areas without family support. Participant no. 11 talked about this unparalleled source of hope:

“At my first course (in rehab camp) my dad and family came to meet me. I kissed my parent’s hands for the first time. They told me we want to keep you here for two courses because we love you. Oh, they told me they loved me. I said, if you want, I’ll stay. When I came back to my room I was laughing. My roommates thought I was nuts. They said: ‘you are told to stay here for two courses and you laugh!?’ I completed my ‘First Step’. My first honesty was that I raised my hand and said I am a drug user. Second, I was finally proud of my parents whereas I used to lie that they were dead. I accept them as they are. I depend on them to stay healthy”.

### B. Sinking factors

Although the participants of the study managed to change, most of them failed to return to their normal lives and, answering our questions, they refer to the following as failure factors “non-assisting mates, pro-addictive family, unawareness of assisting official organization and non-government organization, woman’s lack of authority, and ineffective opportunities”.

Non-assisting mates: Most of the participants lacked family support during the rehab. Participant no. 14 said:

“My family did not help me at all. They did not trust me anymore. They looked at me with hatred. My children and my husband do not still want to see me. It affected me. It made me more determined to use drugs. They wanted me as an addicted person who listened to whatever they said. They did not want to help me. I used drugs, I harmed everyone, I destroyed everything. They did not help me to stop drug use because they could force me. They took me wherever they wanted. They did everything to me: physical abuse, sexual abuse, rational and mental abuse.”

Pro-addictive family: The availability and the use of drugs by all family members at home was another major threat tempting drug use and affecting the rehab-related decision. Participant no. 17 talked about this point:

“I didn’t know what to do. Drugs were found at my mom’s home, and also at my dad’s home, or my sister’s. When I went to my home, my husband used drugs in front of me. I had to use too. I could not refrain, I had a big temptation. I did not want to use it, but I used while crying.”

unawareness of assisting official organization and non-government organization: Lack of awareness of existing society support services or lack of special rehab centers for women were among the most threatening factors mentioned by the participants. For example, participant no. 20 said:

“I was exhausted. I was doing everything to stop drug. I did not know what to do, until one day I was desperately sitting in front of the TV watching a program about rehab centers. I thought that these centers were for men only. It was very late for me. Now that I think, I see that if I went to the rehab camp when I used only opium, the desired result would have come very sooner than when I used several drugs.”

Woman’s lack of authority: In terms of rehabilitation, most of the participants not only lacked financial resources, but also were living as a woman under the supervision of a father, husband, brother or partner. These conditions largely made them fail to stop drugs. These women, even if they had financial resources, had to inevitably stay with their fathers or husbands. Any time their father/husband wanted, the women had to leave the camp. Participant no. 11 said:

“… After 20 times that my husband was taken to the rehab camp, my dad once brought me there to quit. However, my husband gave him 400.000 tomans to bring me back. My husband wanted me to be a drug user, so that I may not think of divorce or making him quit drugs. He gave me subsistence and I had no options for quitting. I was a woman and needed food and a shelter”.

Ineffective opportunities: women drug users participating in the study had gone to rehab camps several times. But they left each time more disappointed than the previous time. The high costs of staying in the camp, lack of permission to leave or reenter the camp, not meeting their children, the absence of a doctor or counselor, lack of physical space and adequate food, and sometimes violent behaviors were factors related to the ineffectiveness of rehab camps. Participant no. 22 stated:

“... The first time at the camp, they were about to beat me. If someone had a pain and was mourning, they shouted at her or slapped her. I was afraid of the other drug users; I was afraid of their looks. It was not good. For example, when eating food, the servants prepared salads but they ate it at the office. They didn’t give any to the drug users. I cried like children; I said if my mom was here, she prepared me salads.”

Participant no. 16 also talked about the conditions of a rehab camp:

“I’m already a postmenopausal woman. I have hypertension and diabetes. I have to take my medicines. But here they did not allow me to take them. One of the women had a bad cold and they did not allow her to take an acetaminophen. They take you out only if you die. It is my last time here.”

Only a few women drug users are able to benefit from outpatient drug quitting facilities. The cost of these centers is not affordable to these women. These conditions, along with getting under the control of men/partners, deprive them of the use of these services. In this regard, participant no. 4 said:

“I went to the camp about 7 to 8 times, but it did not pay off. The last time in the camp, one of the women said: ‘go to the rehab center of Dr. X. He was a professional doctor. But, I could not afford the money they wanted. I tried a lot. I’m not worth more than 50.000 tomans. I wish there was some free of charge rehab centers. I’m exhausted. I’m fed up with trying in vain to quit.”

## Discussion

The participants had different experiences in quitting drugs. Facing several physical, family, social and economic complications caused by drug use is another reason for the recognition of the disaster and, thus, the tendency to quit. Tavakoli (2009) argues in a study that encountering multiple physical, familial, social, and economic consequences of drug use is another reason providing the drug user with a deep understanding of disaster and, thus, a tendency to quit drug [24]. In the study of Alvarez et al., the decision to stop drug use has been made in order to regain balance and health and to make a fundamental change in one’s life [25]. The results of this study also showed that individual factors facilitating decision-making about drug use differ based on one’s gender. The results of the study by Arfken (2001) showed that women drug users suffer more than men from psychological and medical problems, lower professional skills, legal difficulties, lower income, and higher levels of dependency at the time of drug use. They believe that these factors influence the decision of women drug users to quit drug [26]. The results suggested that women drug users have been abused. This for some of the participants was a reason for drug cessation as they became tired of this situation. The results of Newman’s study (2004) showed that social and ethical abnormal behaviors such as prostitution, theft, escape from home and suicide for the preparation of drug have been more prevalent among the female participants than the male ones in the decision for drug cessation. In Newman’s study, women drug users were exposed to the consequences such as sexual, emotional, and physical abuse [27].

Our results also emphasized that all forms of violence (physical, sexual and emotional) are among the factors affecting women’s drug use both in their childhood and adulthood that are in line with the results of Khaledian (2014) [28]. The participants of the study realized their severe conditions when they referred to the compulsive drug use, being threatened, and getting to the end of their rope. These results are also in line with the results of Chang (2017) [29]. In this regard, Stensma (2005) also argues that drug users should be mentally engaged with their problems to figure out their negative conditions and try to escape from it [30]. Doing so, women drug users can gain self-confidence and knowledge, and come to a deep understanding of their conditions to get on their way back to life. Another finding is that, at the pinnacle of helplessness, believing in God’s mercy and resorting to spirituality was a way to return from addiction to life. Research has confirmed the positive role of spirituality at the start of the rehabilitation process and reducing the likelihood of relapse to drug use [31]. In a study, the findings showed the positive results in those who paid attention to proper nutrition, exercise and spirituality in their rehabilitation process [32]. Increasing the internal ability of people, spirituality helps them to find their place in the life in the case of severe stresses. Enhancing compatibility with and resistance to problems, spirituality leads to purposeful life and greater social support among the rehabilitating individuals [33].

In the present study also most of the participants chose a spiritual path to return to a life without drugs under the inspiration of “Steps” introduced by the Association of Anonymous Narcotic. Of course, all of the participants did not feel that way. But those who reconciled with God, that is, with themselves could cope with the temptation of drugs better. However, some of the participants had a different experience and faced with unremitting challenges in the rehabilitation process. As one of the elements of this religious culture, the Iranian drug user has been caught in the trap of drug and, thus, will feel more confident and hopeful through having greater inclination and connection with religion and spirituality. The participants believed that while family has a greater support for the rehab of men, this is not the case for women. Owing to financial dependence or lack of authority in women, they are more vulnerable drug users or are considered to be more threatened subjects. In this regard, many studies as well as the prevention and rehab techniques have always been male-centered. On the one hand, the latest studies have shown that drug dependence is quite different in men and women, which has led to different recommendations for quitting among them [34]. Additionally, women drug users are more likely to enter dependent relationships with their partners than the male ones, and this impedes obtaining the basic life skills such as financial management and future planning. Women drug users have often addicted spouses or partners who not only do not support women in quitting, but also threaten them with violence or relation abandonment [35]. Overall, the participants experienced family support, but most of them were deprived of any support by their family or husband in rehab. According to studies, being rejected by the family and conflict among the family members are among the barriers of sustainable rehabilitation, that the results of the present study complement their findings [14, 36]. On the other hands, family could encourage the person to enter the rehabilitation process [37]. Family can also play an important role in improving the quality of life by providing adequate facilities as well as economic, psychological and emotional support for the subject drug user to motivate drug quitting in them [38]. The common ground between the above studies and the present one is that the role of the family and their support is decisive in entering the drug rehab process. Therefore, recognizing this context and challenges of living with drug users as well as trying to address them is an important requirement in making the decision to quit drug [39]. As such, family counseling for screening psychological disturbances and education through mass media as well as counseling sessions to encourage the drug users’ decision for drug rehabilitation are the necessities which can provide families with the support of these drug users. The use of drugs at home and by all members of the family together with the provision of conditions for the temptation to use drugs was another threatening factor influencing the participants’ decision to quit drug use. According to a study, the main causes of having the tendency to use drug from the viewpoint of women referred to the rehabilitation centers of Kashan city in Iran include an addict family (43%), easy access to drug (64%), depression and hopelessness (54%) [40]. Another study in Tehran, Iran, showed that 56% of drug dependent women had husband drug users [41]. Domino (2005) also found in his study that drug use in the family members contributes to the relapse in the participants [42]. A husband or partner drug user usually gives drugs to his wife and puts her in a dilemma of trust or distrust [19]. In fact, women are involved in drugs more during their intimate marital relationship with their husbands, compared to their other intimate relationships. Male dominance in a relationship seems to act as an unenviable encouragement in women’s addiction [41]. All above mentioned studies are consistent with the results of the present study. Therefore, the necessity is felt for spreading civilians’ culture-based education and women’s empowering to narrow the above-mentioned gap is felt. Regarding the statements of the participants indicating that the proximity to drug users is one of the most important factors in preventing the re-use of the drugs, the existence of safe and suitable treatment centers for women to live in is one of the important factors that need to be considered. The experience of women drug users with the drug rehab camps brought about only more frustration and desperation. Camps were unsuccessful and discouraging. In a study conducted in Chitgar residential-remedial center in Tehran (in Iran), most subjects complained of the severity of the physical complications of quitting and the prohibition of the using any medications during this period. Restlessness, severe physical pain and self-mutilation were reported. The presence of people with a long lasting healthiness history, the presence of a doctor, psychiatrist, counselor and social worker were the most important needs of the subjects [11]. Since the first step in the rehabilitation process is the decision to quit drug, it is necessary to consider gender-related needs and issues of these women in order to increase their motivation for rehabilitation and the continuity of this process through using strategies such as motivational interventions. Women-specific rehab centers could be an important improvement to help women quit drugs.

### Limitation and suggestion

The participants were a special group of women drug users in Iran and, consequently, the findings cannot be generalized to all women drug users in Iran. Further qualitative research on other groups of women drug users in different cities of Iran is recommended. As such, other limitations of the present study were the lack of access to information about participants’ mental disorders before and after drug use and their impact on their experience of quitting. Thus, it is suggested that some research be done regarding the impact of mental disorders on rehabilitation, non-rehabilitation and stability of rehabilitation.

## Conclusion

The results show that the individuals discharged from rehabilitation centers experience numerous problems in different personal, social, economic, and family domains. Issues such as psychological and physical problems, not having effective beliefs, lack of trust and social acceptance, compulsive drug use and concerns for it, low social and economic welfare, poor post-discharge health care and, ultimately, family-related factors, to varying degrees, cause the person to return to drug use. Based on the identified factors, it can be suggested that more counseling and psychological programs be provided for the individuals undergoing drug rehabilitation in order to treat their psychological problems. Teaching life skills to people undergoing treatment, family training sessions for better acceptance of these people after being discharged from the rehabilitation centers, and the reduction of family disputes can be an effective step in reducing the relapse of drug use in these individuals. Consulting with family members to screen for psychological disorders and educating them through mass media and counseling sessions with the purpose of motivating the economic, psychological, and emotional support of the women drug users can play a critical role in the rehabilitation process. Moreover, some social supports, such as providing accommodation for homeless discharged people and providing jobs for them, can also be helpful in reducing the relapse of drug use among these people. The results of the present study can help social planners and those responsible for drug rehabilitation process to understand the supportive factors of drug users as good as possible. By the way, the drug users themselves are their own managers in the process of drug rehabilitation and, thus, awareness of the underlying factors that facilitate the process of a sustainable rehabilitation can provide a good guidance in this regard. Finally, as an element of this culture, the Iranian women drug users may resort to the spiritual values to have a higher hope for a better life.

## Data Availability

The dataset that support the findings of this article belong to the Isfahan Drug Control Headquarters. At present, the survey data are not publicly available but can be obtained from the authors upon reasonable request and with the permission from the Isfahan Drug Control Headquarters. The qualitative data cannot be made available as per the requirement of the Institutional Review Board study approval in order to protect the confidentiality of study participants.

## Abbreviation

HIV: human immunodeficiency viruses
